# Mining Beneficial Genes for Aluminum Tolerance Within a Core Collection of Rice Landraces Through Genome-Wide Association Mapping With High Density SNPs From Specific-Locus Amplified Fragment Sequencing

**DOI:** 10.3389/fpls.2018.01838

**Published:** 2018-12-18

**Authors:** Minghui Zhao, Jiayu Song, Aiting Wu, Tao Hu, Jinquan Li

**Affiliations:** ^1^Rice Research Institute, Shenyang Agriculture University, Shenyang, China; ^2^State Key Laboratory for Conservation and Utilization of Subtropical Agro-Bioresources, South China Agricultural University, Guangzhou, China; ^3^Department of Plant Breeding and Genetics, Max Planck Institute for Plant Breeding Research, Cologne, Germany

**Keywords:** Aluminum tolerance, genome-wide association mapping, relative root elongation, rice landraces, Al tolerant QTL, SLAF-seq

## Abstract

Trivalent Aluminum (Al^3+^) in acidic soils is harmful to root growth and significantly reduce crop yields. Therefore, mining beneficial genes for Al tolerance is valuable for rice production. The objective of this research is to identify some beneficial genes for Al tolerance from rice landraces with high density SNP set from SLAF-seq (Specific-Locus Amplified Fragment sequencing). A total of 67,511 SNPs were obtained from SLAF-seq and used for genome-wide association study (GWAS) for Al tolerance with the 150 accessions of rice landraces in the Ting's rice core collection. The results showed that rice landraces in the Ting's rice core collection possessed a wide-range of variation for Al tolerance, measured by relative root elongation (RRE). With the mixed linear models, GWAS identified a total of 25 associations between SNPs and Al tolerant trait with *p* < 0.001 and false discovery rate (FDR) <10%. The explained percentage by quantitative trait locus (QTL) to phenotypic variation was from 7.27 to 13.31%. Five of twenty five QTLs identified in this study were co-localized with the previously cloned genes or previously identified QTLs related to Al tolerance or root growth/development. These results indicated that landraces are important sources for Al tolerance in rice and the mapping results could provide important information to breed Al tolerant rice cultivars through marker-assisted selection.

## Introduction

Rice (*Oryza sativa* L.) is an important crop in the world. There is about 13% of global rice field on acidic soils (Vonuexkull and Mutert, 1995). Trivalent aluminum (Al^3+^) in acidic soils is harmful to root growth and reduces significantly rice yield (Liu et al., 2012). It is a major toxin for plants on acid soils (Delhaize et al., 2012). Therefore, exploring the genetic mechanism of Al tolerance in rice is of importance to understand why Al^3+^ is toxic to the plants and to breed Al tolerant varieties for rice production.

Serval researches on the genetic mechanism of Al tolerance in rice have been reported (e.g., Famoso et al., 2010, 2011; Cai et al., 2011). Previous researchers have identified a number of quantitative trait loci (QTLs) for Al tolerance in rice (Nguyen et al., 2002; Ma and Furukawa, 2003; Mao et al., 2004; Xue et al., 2007; Famoso et al., 2011), and found a few genes linked to Al tolerance (e.g., Yokosho et al., 2011; Chen et al., 2012; Huang et al., 2012; Xia et al., 2013; Li et al., 2014). In the context of QTL mapping for Al tolerance, most of these previous researches were conducted with bi-parent segregation populations and linkage mapping. Genome-wide association study (GWAS) make it possible to exploit natural genetic diversity and mine beneficial genes in the genome (Zhu et al., 2008). It is important to apply GWAS with modern genotyping technology for QTL mapping for Al tolerance.

In recent years, many QTLs for multiple traits have been identified using GWAS. For example, Huang et al. (2010) conducted GWAS for 14 agronomic traits with high density SNP set and 517 *indica* landraces of rice. Using GWAS with a diverse rice set of 383 accessions, Famoso et al. (2011) found 48 QTLs for Al tolerance, four of which co-localized with previously identified candidate genes for Al tolerance and two of which co-localized with previously identified Al-tolerant QTLs. Using 274 SSR markers and the same populations as this study, Zhang et al. (2016) performed an association study and found a total of 23 QTLs for Al tolerance. However, to our knowledge, no GWAS for rice Al tolerance has been performed using high-density SNPs with a core collection of rice landraces.

Recently, the whole genome sequencing technology is being increasingly used to accurately and rapidly detect numerous variants across the entire genome at the molecular level. The recently developed next-generation sequencing-based genotyping approach, i.e., specific-locus amplified fragment sequencing (SLAF-seq) method is a simplified genome sequencing technology that has shown to be highly accurate and cost-effective (Sun et al., 2013). SLAF-seq has been applied in genetic map construction, QTL mapping, and molecular breeding. However, to our knowledge, no high-density SNPs obtained from SLAF-seq technology has been applied for GWAS in a core collection of rice landraces.

Abundant germplasm resources for Al tolerance are available in the Asian cultivated rice, especially in rice landraces. As early as in 1920–1964, a total of 7,128 accessions of rice landraces had been collected by Prof. Ying Ting, which was named as Ting's rice collection (Li et al., 2011). They were from all over China as well as from some main rice cultivation countries. Based on 48 phenotypic data, Li et al. (2011) has constructed a rice core collection consisting of 150 accessions. The analysis of population structure indicated that there existed two subgroups mainly corresponding to *indica* and *japonica* subspecies and the LD decays to the threshold, i.e., the 95% quantile of *r*^2^ between unlinked loci pairs, at 1.03 cM in the entire collection, which was about 200–500 kb in physical distance (Zhang et al., 2011; Li and Zhang, 2012). The large variation within the core collection provides an important gene pool of genetic diversity and beneficial genes for rice breeding. Therefore, it is worth to perform GWAS with such a core collection for Al tolerance in rice.

The objectives of the study were to (1) perform GWAS for rice Al tolerance to reveal the genetic basis for this complex trait; (2) identify novel functional candidate genes underlying the mapped regions; and (3) to mine the beneficial genes within the Ting's core collection of rice landraces with the newly developed high-density SNP set from SLAF-seq approach.

## Materials and Methods

### Plant Material

The Ting's rice core collection, i.e., a total of 150 accessions of rice landraces were used to screen their Al tolerance (Table S1). The core collection was constructed from 2,262 accessions of 7,128 based on a strategy of stepwise clustering and preferred sampling on adjusted Euclidean distances and weighted pair-group average method using integrated qualitative and quantitative traits (Li et al., 2011). It represents the diversity in the Ting's rice collection. Furthermore, Nipponbare and Xiangnuo 1 (Yang et al., 2007) were chosen as tolerant control and Nante (Fu et al., 2010), Xiangzhongxian 2 (Xu et al., 2004), and IR64 (Khatiwada et al., 1996) for Al sensitive control. These varieties were used to identify an appropriate concentration for Al toxicity.

### Phenotyping for Al Tolerance

The Al tolerance for the 150 accessions of rice landraces were examined according to our previous research (Zhang et al., 2016). To choose an optimal Al^3+^ concentration to screen Al tolerance, the seedlings for two Al tolerant and three Al sensitive rice varieties were exposed to 0.5 mM CaCl_2_ (pH = 4.0) containing 50, 100, 150, 200, 250, 300, 350, 400, 450, and 500 μM AlCl_3_ (no other nutrient solution was applied), respectively. The Al^3+^ concentration under which the largest difference of relative root elongation length (RRE) between the sensitive and tolerant varieties was chosen as an optimal Al^3+^ concentration for screening of Al tolerance in the following experiment. In this case, the largest difference in RRE was observed at 100 μM between the two tolerant and three sensitive varieties. Therefore, the 100 μM AlCl_3_ was used for screening of Al tolerance.

The 150 accessions of rice landraces cultivated at the farm of South China Agricultural University, Guangzhou (23°16N, 113°8E), during late season (July-November) in 2008 and 2009. The seeds were harvested each year. Uniform seeds in each year were surface sterilized in 1% H_2_O_2_ for 30 min and rinsed with deionized water. Then the seeds were put into deionized water at 30°C for 2 days in darkness for germination. The uniform seedlings were transferred to a net floating on a 0.5 mM CaCl_2_ (pH = 4.0) solution in a 1.5 L plastic container. A randomized complete block design (RCBD) with three replicates was applied. Seedlings were grown at 28°C for 48 h before being used for Al toxicity treatment. Then, the seedlings were exposed to 0.5 mM CaCl_2_ (pH = 4.0) containing AlCl_3_ for 24 h, and the root elongation length was measured for each sample. Then RRE was used to evaluate the degrees of Al tolerance of all landraces. RRE was calculated as follows: (root elongation length with Al treatment)/(root elongation length without Al treatment). Root length of 10 seedlings in each treatment was measured before and after treatments. The RRE for each genotype across the three replicates for 2008 and 2009 were calculated, respectively. The mean of RRE for 2 years was also calculated for each genotype. These phenotypic data were used for GWAS.

### Genotyping of SNP Markers

The SLAF sequencing were conducted based on the standard protocol from Beijing Biomarker Technologies Corporation (http://www.biomarker.com.cn) and the introduction by Sun et al. (2013) and Song et al. (2018). To simplify, the first step was to perform a SLAF pre-design experiment with 8 accessions of landraces and different enzymes combinations. This step was used to evaluate the appropriate enzymes and sizes of restriction fragments. The SLAFs obtained by this step should be evenly distributed ascross the genome. The second step was to construct the SLAF library in accordance to the pre-design scheme. In this step, genomic DNA was digested by enzymes designed for individuals. Double barcodes were added to two rounds of PCR reactions to discriminate each individual and to facilitate the pooling of samples. In the third step, the purified DNA tags with indices and adaptors (SLAFs) of 300–400 bp were used and diluted for pair-end sequencing on an Illumina High-seq 2500 sequencing platform according to the Illumina sample preparation guide (Illumina, Inc.; San Diego, CA, US) at Beijing Biomarker Technologies Corporation. All polymorphic SLAF loci were genotyped according to the SNP loci at the reference genome. The SNPs with missing data >20% across all genotypes as well as the SNPs with a minor allele frequency (MAF) (<5%) were excluded for the following statistical analysis. After filtration, 150 accessions of rice landraces with a total of 40,708 polymorphic SNPs were used for GWAS. The data of the SLAF sequencing have been uploaded to the BioSample database (BioSample accession SAMN10448484).

### Statistical Analyses

The statistical model used for GWAS analysis was the PK mixed:$M_{ip}=\mu +a_{p}+\overset{z}{\underset{u=1}{\sum }}D_{iu}\upsilon_{u}+{g_{i}}^{*}+e_{ip}$, where *M*_*ip*_ was the phenotypic value of the *i*th entry carrying allele *p, a*_*p*_ the effect of allele *p, e*_*ip*_ the residual, υ_*u*_ the effect of the *u*th column of the population structure matrix *D*, and $g_{i}^{*}$ was the residual genetic effect of the *i*th entry (Yu et al., 2006; Stich et al., 2008).

Principal coordinate analysis (PCoA) was performed based on all SNPs after filtration. The first and second principal component was used as a *D* matrix of the above-mentioned association approach.

The kinship coefficient *K*_*ij*_ between inbreds *i* and *j* were calculated on the basis of all SNP markers according to:$K_{Tij}=\frac{S_{ij}-1}{1+T}+1$, where *S*_*ij*_ was the proportion of marker loci with shared variants between inbreds *i* and *j* and *T* the average probability that a variant from one parent of inbred *i* and a variant from one parent of inbred *j* are alike in state, given that they are not identical by descent (Bernardo, 1993). For the series of *T*-values 0, 0.025, …, 0.975 **K** matrix between all inbreds was calculated. Negative kinship values between inbreds were set to 0. The optimum *T*-value was calculated according to Stich et al. (2008).

The R package EMMA Kang et al. (2008) was used to perform GWAS. The significance threshold of 0.001 and a false discovery rate (FDR) <10% were applied to test for significant associations between the traits and the SNP markers. The Bonferroni correction was used to adjust false positive rate in the multiple tests (Pocock et al., 1987). The FDR was calculated according to Benjamini and Hochberg (1995). For genome-wide studies with high density SNPs, one must consider the non-independence of SNPs because of linkage disequilibrium (LD) when interpreting statistical significance (Li et al., 2012). To achieve this, we followed Duggal et al. (2008) to randomly select 1 SNP per LD block in addition to all the SNPs outside of blocks. The *p*-values for these SNPs were used for calculation of the FDR. The significantly associated SNPs with Al tolerance within the LD decay distance (i.e., 500 kb from our previous study) was grouped as one QTL. The percentage of genotypic variation explained by the significant SNPs was calculated by $R_{LR}^{2}=1-\exp\left(-\frac{2}{n}\left(\log L_{M}-\log L_{0}\right)\right)$, where exp is an exponential function, log*L*_*M*_ is the maximum log-likelihood of the model of interest, log*L*_0_ is the maximum log-likelihood of the intercept-only model, n is the number of observations (Sun et al., 2010).

### Searching Candidate Genes

To validate our mapping results and find a robust set of candidate genes, we searched the flanking regions ± 500 kb (the maximum LD decay distance in the core collection) of the significant associated SNP loci with Al tolerance to find previously mapped QTLs from the Rice QTL Map database (http://qtaro.abr.affrc.go.jp/qtab/table). Similarly, we searched the flanking regions of the significant associated loci (±500 kb) with Al tolerance to find previously cloned/identified candidate genes related to Al tolerance from the QTARO database (http://qtaro.abr.affrc.go.jp/ogro/table). Because our measurement for Al tolerance was the relative root length with/without Al treatment, i.e., RRE, we think that the genes related to root development are corresponding to Al tolerance. Therefore, we mainly searched the candidate genes related to root development and Al tolerance within the searching regions. Aluminum tolerance genes identified by reverse genetics were found in the OryGenesDB (http://orygenesdb.cirad.fr/cgi-bin/searching.pl).

## Results

The landraces in the Ting's core collection have RRE values ranged from 0.22 to 0.95, indicating a large variation for Al tolerance. The phenotypic distribution of RRE showed a normal distribution, indicating that aluminum tolerance is a quantitively inherited trait. The broad-sense heritability was 88.73% for Al tolerance.

With SLAF sequencing approach, a total of 116,643 high-quality SLAFs were detected, with 24,889 polymorphic SLAF tags and a polymorphism rate of 21.34%. Each SLAF tag had an average coverage depth of 5.2 ×. The inner region of the polymorphic SLAF tags were further sequenced and a total of 67,511 SNPs were detected. After filtering the SNPs with missing data ≧20% across all genotypes and MAF ≦0.05, a total of 40,708 polymorphic SNPs were used for GWAS.

**Figure 1:**
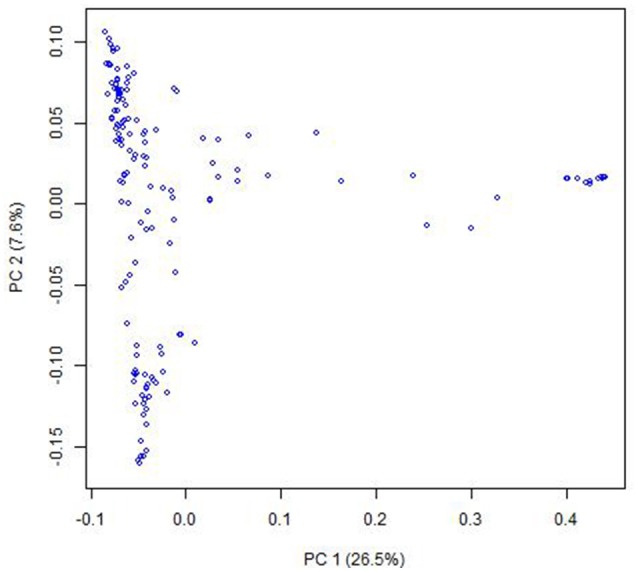
Population structure of the Ting's core collection of rice landraces detected by Principal coordinate analysis. The principal coordinate analysis was based on the entire SNP set for the core collection. PC 1 and PC 2 refer to the first and second principal components, respectively. The number in the brackets indicated the percentage of genotypic variance explained by the principal components.

PCoA indicated that there were two clusters for the entire population (Figure 1), which was corresponding to their classification as *indica* and *japonica* types. Most of the kinship coefficients for any pair of landraces were zero (Figures S1, S2), indicating that these landraces are unrelated, which is due to that they were collected from a world-wide area and were from a core collection. There were also a few pairs of landraces showing high kinship coefficients.

**Figure 2:**
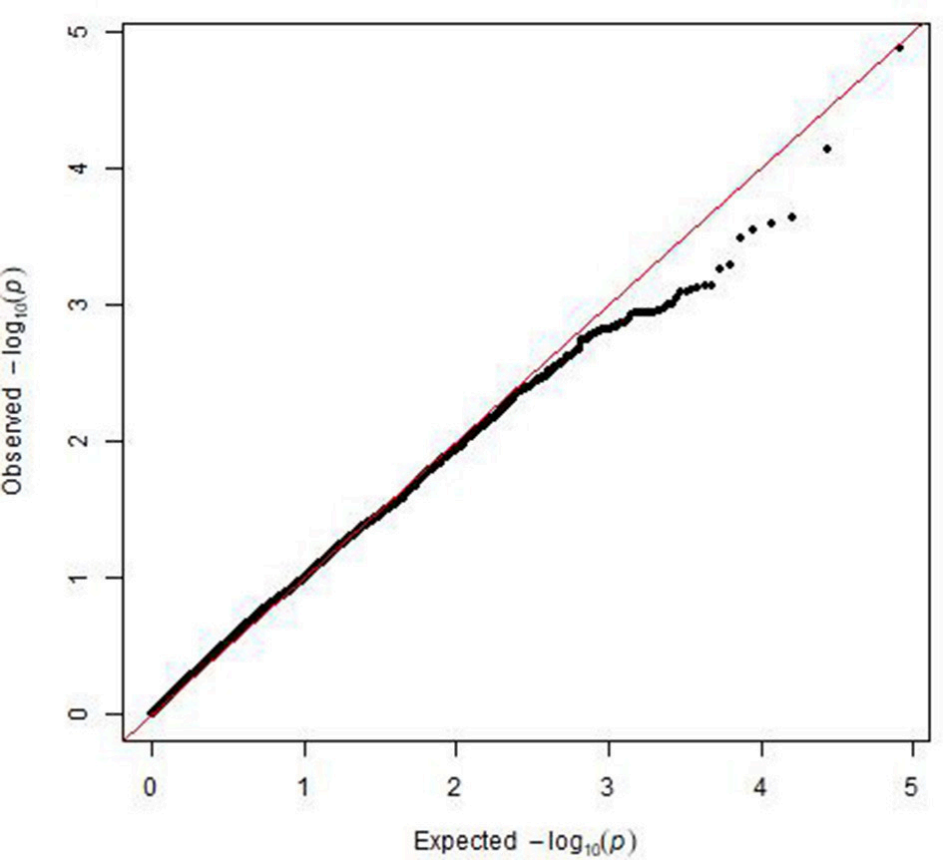
Plot of observed vs. expected *P*-values by using MLM (PK) model for the genome-wide association mapping for aluminum tolerance.

**Figure 3:**
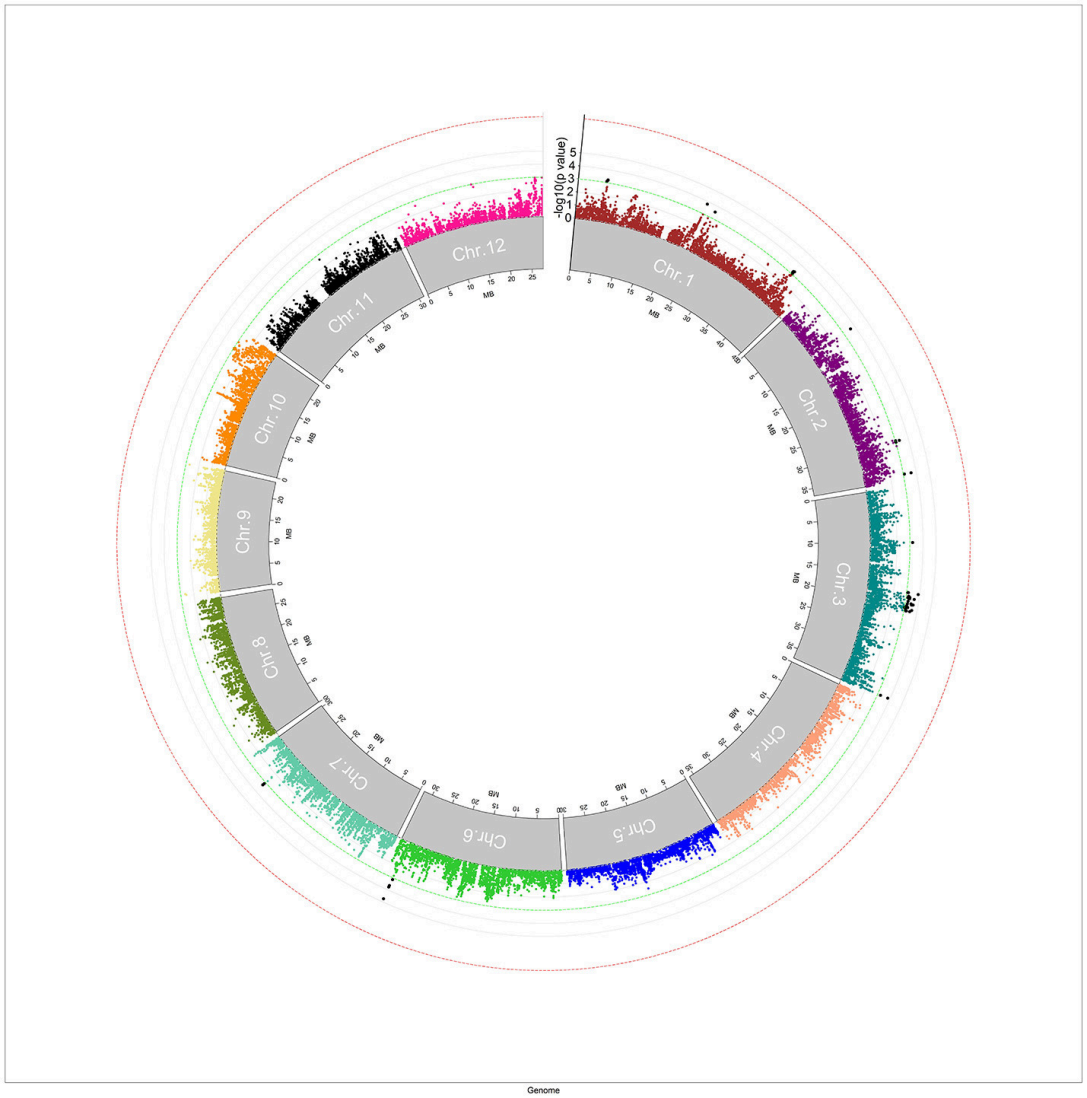
Manhattan plot for genome-wide association study (GWAS) for aluminum tolerance measured by means of relative root elongation length (RRE). The green line indicated the significant threshold at *p* < 0.001, and the red line indicated the Bonferroni significant threshold at *p* < 0.001 (with a raw *p* < 2.46 × 10^−8^).

A mixed linear model, i.e., PK model (Yu et al., 2006; Stich et al., 2008), which accounts for population structure and kinship, was used for GWAS for Al tolerance. To balance the false positive and negative rate, *p* < 0.001 and FDR <10% were used as the significant threshold to indicate whether a SNP was significantly associated with Al tolerance. The QQ plot indicated that the PK model effectively control the false positive (Figure 2). A total of 25 SNP regions were shown significantly associated with Al tolerance (Table 1, Figure 3, Figures S3, S4), but none of them reached the Bonferroni threshold (with a raw *p* < 2.46 × 10^−8^). They were located on chromosomes 1-4, 6-7, 9 and 11. The QTLs explained individually from 7.27 to 13.31% of the phenotypic variance. The fixed effect was ranged from 0.092 to 0.256. There were different number of QTLs on each chromosome ranged from 1 to 7. The number of significantly associated SNPs for each QTL ranged from 1 to 17. Among them, *qALT3.3* and *qALT7.2* were detected in both years data as well as the mean of both years. Most QTLs were detected with the mean of both years data. The beneficial alleles for each significant QTL and their genotype background were further examined (Tables S2, S3).

**Table 1:** Genome-wide association study (GWAS) results for aluminum tolerance and related candidate genes/previously mapped QTLs, where *PV%* is the explained percentage by quantitative trait locus (QTL) to phenotypic variation, FDR is the false discovery rate, and the information on the previously mapped QTLs were from the Rice QTL Map (http://qtaro.abr.affrc.go.jp/ogro/table), the information on the previously clone/identified genes were from the QTARO database (http://qtaro.abr.affrc.go.jp/ogro/table), alumium-activated malate transporter genes were from the OryGenesDB database (http://orygenesdb.cirad.fr/cgi-bin/searching.pl).

**QTLs**	**Chr**.	**Position (bp)**	***P*-value**	***PV*%**	**Fixed effect**	**No. Significant SNPs**	**Previously mapped/clone genes**	**Closest distance to QTLs in this study (kb)**	**Previously mapped QTLs**	**Closest distance to QTLs in this study (kb)**	**FDR(%)**
*qALT1.1*	1	5,170,120	0.000255	9.64%	0.128	10					8.51%
*qALT1.2*	1	11,375,341	0.000346	10.03%	0.153	6					9.33%
*qALT1.3*	1	13,652,501	7.38E-05	11.64%	−0.143	2					7.02%
*qALT1.4*	1	22,591,416	0.00016	10.81%	−0.146	3					6.54%
*qALT1.5*	1	24,383,999	0.000298	10.04%	0.119	2					6.54%
*qALT1.6*	1	41,150,101	9.27E-06	13.26%	−0.243	17	*OsFRDL4*	433.58	(Wu et al., 1999, 2000; Mao et al., 2004)	108.94	8.51%
*qALT2.1*	2	9,045,415	0.000952	7.89%	−0.165	1					9.40%
*qALT2.2*	2	29,749,048	0.000558	8.78%	0.231	5	*Os02g49790.1*	264.06			8.51%
*qALT2.3*	2	35,731,595	0.000735	8.56%	0.207	2					6.54%
*qALT3.1*	3	9,890,146	0.000986	7.76%	−0.128	1	*OsApx1*	3.85	(Zhang et al., 2016)	60.15	8.51%
*qALT3.2*	3	18,562,071	1.33E-05	13.31%	−0.150	6					6.54%
*qALT3.3*	3	19,197,531	7.22E-05	11.28%	0.256	11					6.54%
*qALT3.4*	3	20,589,044	0.000249	9.16%	−0.092	10					6.54%
*qALT3.5*	3	21,188,740	0.000129	11.44%	0.178	14					6.54%
*qALT3.6*	3	28,065,043	0.000574	8.29%	−0.142	2					9.33%
*qALT3.7*	3	36,959,662	0.000354	9.18%	0.164	2					6.54%
*qALT4.1*	4	1,751,622	0.000655	8.99%	−0.155	1					2.22%
*qALT4.2*	4	31,480,910	0.000235	9.85%	−0.172	2					2.22%
*qALT6.1*	6	27,430,269	0.000298	9.62%	0.171	3					9.40%
*qALT6.2*	6	30,477,271	0.000257	10.32%	−0.173	13	*STAR1*	449.75			2.15%
*qALT7.1*	7	8,865,818	0.000665	8.69%	0.172	2					9.40%
*qALT7.2*	7	25,216,755	0.000148	10.25%	−0.111	6					8.51%
*qALT9.1*	9	23,500,139	0.000229	9.60%	0.105	2					3.20%
*qALT11.1*	11	4,955,718	0.001512	7.27%	0.126	9			(Xue et al., 2007)	446.02	9.40%
*qALT11.2*	11	17,203,545	0.000448	9.48%	−0.135	1					9.40%

To validate the mapping results, two databases, i.e., QTARO database and OryGenesDB, were used to screen the previously cloned genes and mapped QTLs around the flanking regions ±500 kb of the significant associated SNP loci with Al tolerance. A total of three QTLs mapped in this study were mapped to the same regions as the previously mapped QTLs for Al tolerance (Table 1). The closest distance for the previously mapped QTLs to the QTLs in this study ranged from 60.15 to 446.02 kb.

A total of three QTLs in this study were co-localized with the previously clone/identified genes (Table 1), including the well-known Al tolerance gene *STAR1*. The candidate genes functions include Al tolerance, root growth, root development (e.g., root length, elongation, crown root). The closest distance for the candidate genes to QTLs in this study ranged from 3.85 to 449.75 kb.

Furthermore, one Al tolerance genes identified by mutation analysis from previous research, i.e., *Os02g49790.1*, was co-localized with the QTLs in this study (Table 1). The distance for the gene to the QTLs in this study is 264.06 kb. The gene has the function as aluminum-activated malate transporter or aluminum resistance protein.

In total, five of 25 QTLs identified in this study were co-localized with the previously cloned genes or previously identified QTLs related to Al tolerance and root traits.

## Discussions

Asian cultivated rice (*Oryza sativa*) was domesticated from its wild relative *O. rufipogon* (Kovach et al., 2007; Sang and Ge, 2007). Because of domestication and artificial selection of rice, genetic diversity has been remarkably reduced in many cases, and favorable alleles or genes might have been lost in the modern cultivars. Rice landraces are the intermediate form between modern cultivars and their ancestral species. Because of the less impact by artificial selection, landraces contain abundant genetic diversity and useful beneficial genes for modern cultivars. Moreover, transfer of beneficial genes from the intermediate forms to modern cultivars is considerably easier than from the ancestral wild species. Therefore, the identification and utilization of valuable genetic resources in landraces can be highly valuable for the genetic improvement of modern rice cultivars, for example, breeding varieties for Al tolerance.

The Ting's core collection of rice landraces is one of the earliest rice collection in China. Our previous studies indicated that two subgroups were presented in Ting's core collection, corresponding to *indica* and *japonica* subspecies (Zhang et al., 2011). Association studies were performed with 274 SSR markers for important agronomic trait and Al tolerance (Zhang et al., 2014, 2016), which confirmed that the core collection is a good population to map natural variations existing in the rice landraces. Compared to this study, a total of three QTLs, i.e., *qALT1.1, qALT3.1*, and *qALT3.2*, were identified in both our results and the previous research of Zhang et al. (2016). The closest distance for the QTL of Zhang et al. (2016) to the QTLs in this study ranged from 60.15 to 623.69 kb. In addition, there were three QTLs in the research of Zhang et al. (2016) having a distance between 1.7 and 2 Mb (about 8 cM) to the QTLs in this study. However, the mapping results were limited by the numbers of markers used in the previous studies. Because of its high-throughput and cost-effective nature, SLAF-seq is an ideal method for genotyping by sequencing and hence has been applied in this study. This method allowed us to obtain a total of 67,511 high-quality SNPs, which provide good foundation for our GWAS in this study.

In this study, we detected a total of 25 QTLs for Al tolerance (Table 1). The significant associations were distributed 8 of 12 chromosomes in rice, which was in accordance with the research of Famoso et al. (2011). The QTLs explained individually from 7.27 to 13.31% of the phenotypic variance in this study, which was smaller than those in research of Nguyen et al. (2003) and Famoso et al. (2011), while it was larger than that in research of Xue et al. (2007). This might be explained by different mapping populations used in the aforementioned studies and the total phenotypic variation were different. A total of 5 from 25 QTLs in this study were co-localized with the previously cloned genes or previously identified QTLs related to Al tolerance and root traits (Table 1), which was similar with the research of Famoso et al. (2011).

Huang C. F. et al. (2009) and Huang X. H. et al. (2009) found two genes, i.e., *STAR1* and *STAR2*, responsible for Al tolerance in rice. The QTL *qALT6.2* was co-localized with *STAR1* with a minimum distance of 449.75 kb. Moreover, *qALT6.2* was also co-localized with other two candidate genes *OsHMA2* and *OsPTR9* with a minimum distance of 61 and 237.92 kb, respectively, which have the function of Zn and Cd translocation, and lateral root formation.

Furthermore, the *qALT1.1* in this study located at 938.62 kp away from the Al tolerance gene *OsCDT3*, which was identified by knockdown method (Xia et al., 2013). This QTLs was at 623.69 kb away from a previous mapped Al tolerance QTL (Zhang et al., 2016). The *qALT1.6* in this study was co-localized with a candidate gene, i.e., *OsFRDL4* with a distance of 433.58 kb (Table 1). *OsFRDL4* is an Al tolerance gene identified by mutant method (Yokosho et al., 2011). Moreover, this QTL was co-localized with QTLs for Al tolerance identified by some previous researches (Wu et al., 1999, 2000; Mao et al., 2004) (Table 1) with a minimum distance of 108.94 kb.

The QTL *qALT2.2* was co-localized with an aluminum-activated malate transporter gene (*Os02g49790.1*) with a minimum distance of 264.06 kb (Table 1). A previous mapped QTL associated with arsenic accumulation was located 228.11 kb away (Zhang et al., 2008), which implied that the mechanism of tolerance to metal ion (for example, Fe, As, Zn, Cd, etc) might have a similar metabolism way. The explanation could be supported by the observation that several candidate genes co-localized with the QTLs in this study have the functions on Fe and Cadmium uptake, Zn and Cd translocation, etc. The QTL *qALT3.1* was co-localized with an Al tolerant QTL (Zhang et al., 2016).

It is interesting that the regions between 18,562,071 and 21,188,740 on chromosome 3 showed several peaks (the number of significant SNPs ranged from 6 to 14), corresponding to *qALT3.2, qALT3.3, qALT3.4*, and *qALT3.5*, significantly associated with Al tolerance in this study. However, only six candidate genes were identified within this region from the *QTARO* database and only one candidate gene was related to Cadmium and Iron uptake. No candidate genes were identified to associate with Al or other metal ion tolerance. As the mapping results in this study were highly significant, this region as well as other QTLs (*qALT1.2, qALT1.5, qALT2.3, qALT3.6, qALT3.7, qALT4.1, qALT6.1*, and *qALT9.1*) in this study where no candidate genes/previous mapped QTLs were found, could be new loci for Al tolerance and required further research.

## Author Contributions

JL and MZ designed the study. MZ, JS, AW, and TH performed SLAF-seq experiment and data analyses. JL performed GWAS and statistical analyses. MZ, JL, and JS performed searching candidate genes/QTLs. JL wrote the paper. All authors read and approved the final manuscript.

### Conflict of Interest Statement

The authors declare that the research was conducted in the absence of any commercial or financial relationships that could be construed as a potential conflict of interest.

## Supplementary Material

Figure S1 Kinship distribution for the landraces in the Ting's core collection.

Figure S2 Kinship distribution for the landraces in the Ting's core collection, where the kinship coefficient equal to zero were excluded.

Figure S3 Manhattan plot for genome-wide association study (GWAS) for aluminum tolerance measured by relative root elongation length (RRE) in 2008. The green line indicated the significant threshold at *p* < 0.001, and the red line indicated the Bonferroni significant threshold at *p* < 0.001 (with a raw *p* < 2.46 × 10^8^).

Figure S4 Manhattan plot for genome-wide association study (GWAS) for aluminum tolerance measured by relative root elongation length (RRE) in 2009. The green line indicated the significant threshold at *p* < 0.001, and the red line indicated the Bonferroni significant threshold at *p* < 0.001 (with a raw *p* < 2.46 × 10^−8^).

Table S1 Variety names, origin, and classification of *indica*-*japonica* for the 150 accessions of rice landraces in the Ting's core collection, where TI, IC, TJ, JC represent typical *indica, indica*-clined, typical *japonica, japonica*-clined rice, respectively.

Table S2 SNP data for significant QTLs for each genotype with their relative root elongation length (RRE) values.

Table S3 Allelic effect for the significant QTLs.
