# Supplementary figures and images for: Mining Beneficial Genes for Aluminum Tolerance Within a Core Collection of Rice Landraces Through Genome-Wide Association Mapping With High Density SNPs From Specific-Locus Amplified Fragment Sequencing
Source: Front Plant Sci. 2018 Dec 18;9:1838. doi: 10.3389/fpls.2018.01838 (PMC6305482; doi:10.3389/fpls.2018.01838)

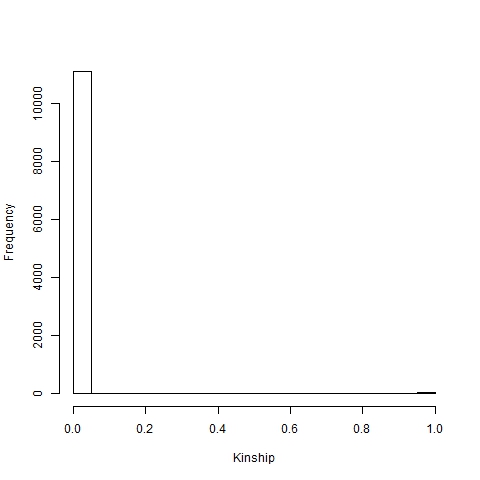

Supplement: Figure S1 — Kinship distribution for the landraces in the Ting's core collection. [file Image_1.JPEG]

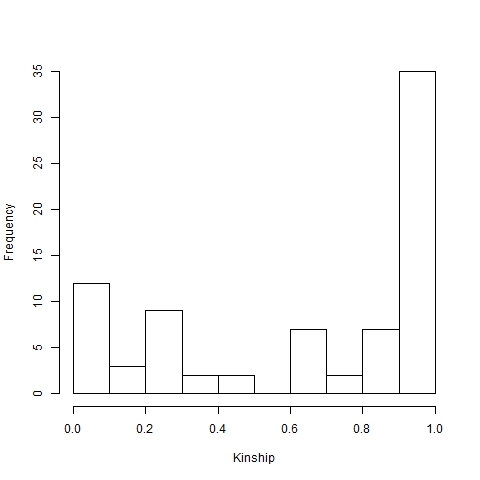

Supplement: Figure S2 — Kinship distribution for the landraces in the Ting's core collection, where the kinship coefficient equal to zero were excluded. [file Image_2.JPEG]
